# An analysis of systemic incident investigation methodologies applied in serious injury or fatality events: A rapid systematic review

**DOI:** 10.1016/j.puhip.2025.100598

**Published:** 2025-02-19

**Authors:** Joanne E. Porter, Elissa Dabkowski, Warren Smith, Alex Fernando, Liz Seaward

**Affiliations:** a Director of the Collaborative Evaluation and Research Centre (CERC) Federation University Australia, Gippsland, Victoria, Australia; b Research Fellow Collaborative Evaluation and Research Centre (CERC) Federation University Australia, Gippsland, Victoria, Australia; c Incident Analytics® Pty Ltd, Australia; d Collaborative Evaluation and Research Centre (CERC) Federation University Australia, Gippsland, Victoria, Australia

**Keywords:** Fatalities, Causation factors, Safety, Serious injury and fatality potential

## Abstract

**Objective:**

This rapid review examines studies that have applied an accident causation analysis method building upon the seminal systematic review conducted by Hulme et al. (2019).

**Study design:**

A rapid review of the literature.

**Methods:**

The following databases, Scopus, EBSCO, Academic Search Compete, CINAHL Complete, MEDLINE, APA PsycArticles, APA PyscINFO, Business Source Ultimate, Business Source Complete, Web of Science and Science Direct were searched for articles that were published from 2019 to June 2023. Eligible studies applied accident analysis modelling to serious injury and fatalities across a variety of industries.

**Results:**

A total of five papers met the inclusion criteria of the rapid review. The studies applied a variety of accident causation models from single large-scale accidents to multiple accident analysis originating predominately from manufacturing industries. The data continued to support the evidence of accident causation analysis models focus on errors, malfunctions, and deficiencies rather than a whole of systems approach and remained complex and difficult to interpret. Based upon the core elements of existing models and following the rapid review of the literature, a novel accident causation analysis approach called the SCALE® Process Model was introduced.

**Conclusion:**

There is need to further explore research-based incident analysis reporting systems that can be applied across a variety of industries and disciplines. The SCALE® Process Model uses systemic techniques to provide a deeper understanding of how multiple factors contribute to the severity of an event aiding in reducing the incidence of serious injuries and fatalities.

## 1 Introduction

Accident causation and incident investigation methodologies vary from linear approaches through to contemporary systems approaches, developed to better address the increasingly complex work environment. The foremost contemporary methodologies continue to include AcciMap [2], STAMP (Systems Theoretic Accident Model and Process) [3], FRAM [4] (Functional Resonance Analysis Model) and HFACS [5] (Human Factor Analysis and Classification System). These methodologies apply a systems view of accident causation and have been applied in many contexts including mining, aviation, health and transport [1]. While these methodologies continue to be at the forefront of safety investigation, it is important to note that they are often difficult and complex to apply and may need to be evaluated for their continued efficacy with emerging hazards and technologies.

Traditionally alarm-based approaches have been used to alert the operator to a risk or potential danger. This approach relies on the reaction and capability of the operator to respond to the alarm and then act to reduce or eliminate the risk, returning operations to a state of safety [21]. With advances in technology and the introduction of Remote Operation Centres (ROC’s), as seen in offshore industries such as gas and wind, operators are required to develop an enhanced situation awareness and capabilities for early interventions to prevent unwanted events and incidents [21]. It is imperative that operators are provided with comprehensive knowledge and awareness of early sense-making of deviations. It is therefore important to combine suitable technologies, with practical modelling towards enhancing human expertise in early perception and interpretation of deviations in addition to prevention and prediction of unwanted events.

In a study conducted by Omidi et al., which aimed to prioritise the influential factors (including safety climate, perceived organisational support for safety, perceived supervisor support for safety, safety voice, organisational resilience and individual resilience) affecting healthcare workers safety performance in a women’s hospital [22]. This study highlighted the need to consider all staff across an organisation from different disciplines and with different roles and responsibilities to fully assess an organisations safety performance.

Rasmussen (1997) developed the risk management framework based on his argument that higher-level decisions in organisations can impact on health and safety by influencing what occurs at lower levels [6]. The framework also identifies the importance of communicating relevant information up the hierarchy from workers and others to help shape those higher-level decisions. The AcciMap [7] levels include government, regulators and associations, the organisation, management, staff and the work itself. The process provides a way to graphically map those conditions which may have contributed to an incident, by detailing the elements, decision-making and actions as well as the relationships between each of these levels in the framework. Svedung and Rasmussen’s (2002) main aim in developing the AcciMap was the “control of hazardous process at the bottom of the socio-technical system.” (p. 406). The AcciMap therefore assists in analysing the vertical interactions and relationships between levels, to improve the systems and their impact on workplace health and safety.

Leveson’s (2004) STAMP begins with the AcciMap methodology and builds on this with an increased focus on upstream processes to help prevent the conditions for the incident, and the control aspects of the system over and above information flow. STAMP also includes a classification of incident factors. STAMP views incidents as a problem or failure of control within the system. Incidents may occur when the control system cannot sufficiently resolve failures in components, ineffective interactions between components and/or external disruptions. “Safety … can be viewed as a control problem” (p. 250) where the control structure was not effective in reinforcing appropriate system constraints. In STAMP the system is a set of interrelated components maintained in equilibrium by feedback loops. Safety management, therefore, is a continuous process of maintaining system constraints to restrict the system’s behaviour in response to changes and adaptations. Incidents can be investigated by understanding why the constraints and controls were insufficient.

The FRAM [4] presents a way to explain the everyday variability of work performance and the concept of resonance. FRAM is based on four underlying principles.

• “Failures and successes are equivalent in the sense that they have the same origin” (p. 22). Put simply, each time a task is undertaken the outcome may be different. This does not mean that the underlying process has changed.
• Conditions for work are never exactly the same as how the work was prescribed to be carried out. Individuals and groups will make adaptations in their performance to meet the local conditions. This is also known as work as done (WAD) versus work as imagined (WAI) when local practices are different to the formal, documented policies and procedures [8].
• Emergence, the third principle, is an outcome of the variability of multiple functions which may combine in unexpected ways which leads to outcomes with non-linear effects.
• The final principle, functional resonance refers to the overall approximate adjustments people, individually and in groups, make to get work done every day. While seemingly random, these adjustments have a level of predictability, based on their overall function. Every function has variability. Variability comes from humans, technology, barriers and/or latent conditions. Once the variability of the overall system becomes too much for the system to absorb it generates an unwanted and undetectable outcome, an accident.

A FRAM is conducted by analysing and mapping a set of work activities or a work process by describing the functions required to carry out the work and their connections with each other.

The HFACS [5] methodology utilises Reason’s Swiss cheese model and includes categories and classification taxonomies which provide additional information [9]. HFACs introduced definitions for the latent and active failures identified in Reason’s Swiss cheese model. Initially this classification was developed based on analysis of military aviation accidents. There are four levels of failure in HFACs, corresponding to the four layers within Reason’s model. These four levels are:

• Unsafe acts of workers includes errors and violations. These are the human activities that fail to meet the expected outcomes.
• Preconditions for unsafe acts. This includes identifying the condition of the worker, environment factors and personal factors
• Unsafe supervision. Four categories of unsafe supervision have been identified in the HFACS. These are inadequate supervision, planned inappropriate operations, failures to correct a known problem and supervisory violations.
• Organisational influences. Decisions made higher up the organisation can directly impact at lower levels These factors can include resource management, organisational culture and organisational processes.

Each of these levels can be broken down further to define the “holes in the Swiss cheese” (p.70). The HFACS can be applied by working back from the accident and the immediate causal factors to classify the errors and causal factors using the classifications available [9]. The four accident methodologies outlined above have encouraged a complex systems perspective on accident causation. As the complexity of emerging hazards and technologies continue, these methodologies may need to be reimagined to keep pace with the change to systems thinking.

Hulme et al. (2019) conducted a systematic review of systems thinking accident causation analysis methods to better understand the causes of accidents in a range of sociotechnical systems context. A total of 73 articles were included in the review, exploring the four method categories, AcciMap, HFACS, STAMP-CAST and FRAM. Table 1 provides a summary of the four categories, describing the focus, elements, and limitations of each method.

**Table 1:** Summary of the current four method categories.

Method	Focus	Elements	Limitations
AcciMap Accident analysis	Risk management framework	Government policy, regulators, company management, technical and operational management, physical processes and actor activities staff and equipment and surroundings.	Focus on errors, failures, malfunctions and deficiencies
HFACS Developed for aviation sector	Latent and active failures	Four levels, unsafe acts, preconditions for unsafe acts, unsafe supervision, organisational influences	Focus on errors, failures, malfunctions and deficiencies. Lacks a clear quantitative measure.
STAMP-CAST Developed for space, aviation, medical, defence and automotive sectors	Systems theoretic process analysis and accident analysis	Considers safety as a control issue, managerial, organisational, operational, manufacturing-based and social controls.	Focus on errors, failures, malfunctions and deficiencies
FRAM Developed for aviation sector	Risk and hazard analysis	System functions, human, technology, organisational or other. Six aspects, input, output, precondition, resource, time and control.	Outputs can be highly complex and difficult to interpret

Insert - Table 1 Summary of the current four method categories.

Currently the most widely used method is HFACS (n = 43) followed by AcciMap (n = 20), STAMP-CAST (n = 6) and FRAM (n = 4)^1^. Hulme et al. (2019) concluded that there was a need to upgrade incident reporting systems and further explore innovative new ways to develop novel accident causation analysis approaches. Advanced technologies and emerging new industries will require new and innovative incident reporting systems to prevent unwanted events and incidents. A model that can transverse industries and enhance cross learning will be better situated to improve organisational and individual sense-making of early deviations. The existing models, lack transferability across the breadth of complex industries and can be therefore difficult to apply. This paper aimed to review the lessons learnt from the four method approaches and introduce a new model that incorporates advanced elements and analysis software to review fatal and serious injury accident causation to better inform practice and systems change.

## 2 Aim

The overarching aim of this rapid review was to explore the various systemic methodologies used to analyse serious injury or fatality events across workplace industries. This review seeks to examine recent literature and identify research gaps that would benefit from further inquiry.

## 3 Method

### 3.1 Design

A rapid review was chosen as the preferred typology for this review. A rapid review is characterised by the simplification of the traditional steps in a systematic review, but still provides an effective, transparent and rigorous evidence synthesis [10]. Given the high quality review by Hulme et al. (2019), the authors opted to conduct a rapid review with a five year date range to investigate any advances in the literature.

### 3.2 Search strategy

In conjunction with a research librarian, a review protocol was devised and prospectively registered on Figshare [11]. The search was conducted on March 29, 2022 using the following electronic databases: Scopus, EBSCO (Academic Search Complete, CINAHL Complete, MEDLINE, APA PsycArticles, APA PsycINFO, Business Source Ultimate, Business Source Complete), Web of Science and Science Direct. A Boolean and truncation search strategy was devised using key search terms (see Table 2). These search terms were also guided by those used in Hulme et al. (2019). Limiters were applied to include full-text, peer-reviewed studies published in the English language between the years 2017–2022. The authors also conducted a manual search using Google Scholar and reference lists to identify additional studies. All citations were uploaded to Endnote and transferred to Covidence to facilitate collaboration between authors [12].

**Table 2:** Example of search strategy.

Search Strategy
“systems thinking accident analysis methods” OR “system methodology∗” OR AcciMap OR “Human Factors Analysis and Classification System OR HFACS OR “Systems Theoretic Accident Model and Processes OR Causal Analysis OR Functional Resonance Analysis Method OR FRAM OR socio-technical systems
workplace OR work∗ OR occupation∗ OR industr∗
analysis OR investigat∗ OR evaluation OR review
fatalit∗ OR "serious injur∗" OR death

### 3.3 Screening and eligibility

Duplicate records were removed in Covidence, and authors subsequently completed a title and abstract screen independently. As per the protocol the authors originally intended to search for primary articles only, however this search was extended to include high quality reviews as well. Articles were included if they used systemic methodologies to analyse industrial accidents that resulted in serious injuries or fatalities. Therefore, studies were excluded from this rapid review if they focused on the use of non-systemic modelling or focused on near-miss accidents. The approved screened records were obtained in full-text and further evaluated to determine eligibility by two independent authors. If consensus was not reached, a third author moderated the selection process. The final list of studies included in this rapid review are unanimously approved by all authors.

### 3.4 Quality appraisal

The included papers underwent a critical appraisal process using tools recommended by the Joanna Briggs Institute (JBI). The purpose of conducting a quality appraisal is to cogitate the methodological quality and the risk of potential bias [13]. The tools used for this process were the JBI Checklists for Systematic Reviews, Cohort Studies, Qualitative Research and the Checklist for Text and Opinion, pursuant to the study design [13]. The quality appraisal was conducted by two researchers independently and a summative score was detailed in the data summary table. This rigorous appraisal can be used to inform the synthesis and interpretation of the findings [13].

### 3.5 Data Extraction and analysis

The data from the included studies was extracted verbatim and presented in a data summary table. The information from each article included citation details, study aim, study design, population/industry and key findings, Table 3). The data was independently extracted and co-verified by two authors. A narrative summary of the findings was undertaken with the intent to add to the seminal review of the literature conducted by Hulme et al., 2019 to inform the development of a new accident analysis model.

**Table 3:** Summary of key findings.

Author, year, country	Accident Causation method	Study aim	Study design	Population/industry	Key findings
Woolley, M. Goode, N. Read, G. & Salmon, P. 2018 Australia	AcciMap	To apply the AcciMap technique to the construction industry to analyse the contributing factors to prevent incident reoccurrence.	AcciMap technique was applied to accident investigation reports to analyse the contributing factors in the reports. 100 serious injury or high potential accident investigation reports from five Australian construction organisations.	Three medium-sized (100–500 employees) and two large organisations (501–2000 employees) in the Construction industry. Used Incident Causation and Accident Analysis method (ICAM)	AcciMap has not been applied in the construction context. Seven levels were identified: Government, regulatory bodies, client and external associations, company management, operational management, staff and work. Reports focused on human error and decision-making factors. Need to also focus on understanding accident and causation from a systems perspective.
Filho, A. Berlink, T & Vasconcelos, T. 2019 Brazil	HFACS	To analyse the causal factors of machine and equipment accidents from 2009 to 2014 in Brazil.	Four analysts coded each incident/accident case following a training using the HFACS framework. Cases were coded independently.	Accident reports from the Ministry of Labour classified as industrial activities involving machines and equipment.	A total of 96 accident casual factors were coded in active errors, preconditions for active errors and unsafe supervision. Unsafe acts were identified in 90.5 % of cases. Unsafe supervision was identified in 87.1 % of cases. Organisational influences were identified in only 56 % of cases.
Woolley, M. Goode, N. Read, G. & Salmon, P. 2019 Australia	Cross section of models	To determine if construction accident analysis literature has applied a systems thinking approach to understand accident causation.	A literature review of the three types of accident causation models, simple linear, complex linear and complex non-linear models.	Construction sector	Three types of accident causation models. Simple linear – culmination of predictable preventable series of events. Complex linear – interaction of underlaying latent conditions and unsafe human acts for example the Swiss Cheese Model. Complex non-linear – system-wide factors and complex interactions between individuals, organisations, technology, behaviours, performance and safety. Identified 266 contributing factors. There was a deficit in systems thinking. An absence of identification or examination of regulatory factors. Lack of focus on higher level factors.
Yousefi, A. Hernandez, M.R. & Pena, V.L. 2018 Spain	Comparison Between AcciMap, FRAM and STAMP	To compare and discuss the difference in using FRAM, STAMP and AcciMap to analyse a single accident.	Case study comparison of three accident analysis models used to analyse the Chevon Richmond refinery accident which was investigated by the U.S. Chemical Safety and Hazards Investigation board.	In 2012 the Chevon Richmond refinery experienced a catastrophic pipe rupture of light gas oil. Following the accident 15,000 people in the area sought medical attention.	AcciMap was used officially while FRAM and STAMP were applied to the Chevron accident retrospectively. The results indicated that the STAMP model was more instrumental and comprehensive in generation of recommendations because it captured failures, inadequate controls, unsafe decisions, control actions and process model flaws. STAMP provided a more complete understanding of the causes of the accident, enhancing the ability to identify system improvements.
Marquardt, N. 2019 Germany	Situation Awareness (SA)	To test the assumptions of the triple-loop learning model of human error and SA in a sociotechnical real-world manufacturing work environment.	Teams of 15–20 people rotated to simulated marked workstations in the production line and answered the situation awareness performance test and the human error questionnaire.	A total of 108 employees of a large automotive manufacturing company were tested using the situation awareness performance test (SAPT) and the human error questionnaire.	The human error questionnaire was based on the “Dirty Dozen Errors” originating from aviation maintenance and covers the 12 most common causes of human error. The study found that having an appropriate mindset of error causation is a prerequisite for organisational learning and maintaining adequate situation awareness. This simulated study was in a single industry and would need further testing to assess its application in other sectors.

## 4 Findings

**Fig. 1:**
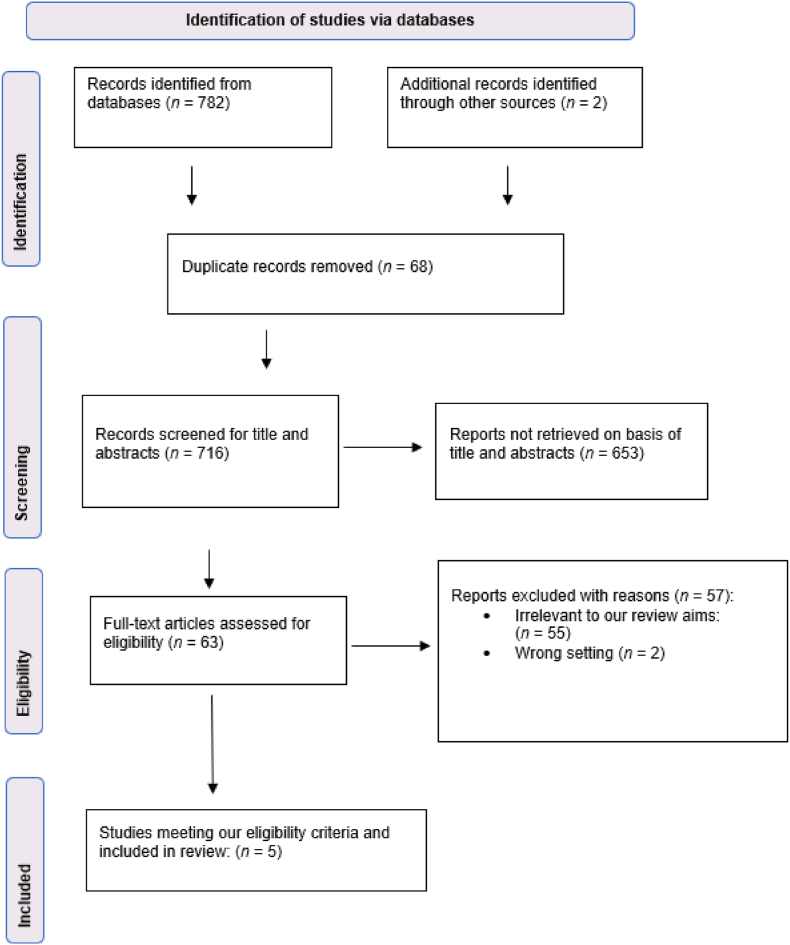
Modified PRISMA flowchart of the search strategy (Page et al., 2021)

A total of 782 records were identified from the database search. Following a modified PRISMA search strategy systematic approach (Fig. 1), five papers met the inclusion criteria. The included papers selected further examine the use of causation models following on from the work of Hulme et al. (2019) who explored the use of applied systems thinking accident analysis methods in a diverse range of sociotechnical systems contexts. The five additional peer reviewed studies helped to further understand the application and limitations of each of the current models.

There were two studies conducted in Australia both from the construction sector, firstly applying the AcciMap technique across 100 serious injuries or high potential accidents. This was the first time AcciMap had been applied in the construction context. The study highlighted the need to also focus on understanding accident and causation from a systems perspective [14]. The authors went on to explore a cross section of models that could be applied in the construction sector describing the types of models from ‘Simple linear’ which focuses on the predictable elements of an accident, ‘Complex linear’ which applies the Swiss cheese model approach and the ‘Complex non-linear’ which reviews factors system wide [15]. There was a lack of focus however on higher level factors which did not consider regulatory factors.

A study conducted in Brazil reviewed 96 machine and equipment accidents from 2009 to 2014 that were reported to the Ministry of Labour. The HFACS method was applied by four analysts who coded each of the cases, identifying that unsafe or inadequate supervision was a contributing factor in 87.1 % of the cases [16]. Using four independent analysts enabled for testing of reliability and confirmability of the method. In comparison, a study conducted in Spain compared FRAM, STAMP and AcciMap on a single large incident which resulted in mass casualties and injuries. In this instance STAMP was the method that produced the most comprehensive and useful information because it captured failures, inadequate controls, poor decision making and process systems flaws [[17], [18]]. There remains method uncertainty, resulting in multiple models being applied simultaneously to assess all the elements of a case further demonstrating the gaps in each of the existing models to be applied across sectors and across all causation elements.

A study by Marquardt (2019), highlighted the importance of situation awareness in testing for human error in a sociotechnical manufacturing environment in Germany. Using simulated workstations to represent a large automotive manufacturing production line process employees were tested using the Situation Awareness Performance Test (SAPT). The study highlighted the need to include situation awareness into existing models as an important element in order to understand organisational learning, however further testing would be needed to assess its application in other sectors [19].

The included five studies, combined with the work of Hulme et al. (2019) continue to highlight the limited application of current accident causation models and the focus of human or mechanical error with an inability to also consider systemic factors. The need for a fit for purpose model that can be applied across organisational sectors led to a further review of the current available models, and the subsequent development of a new model informed by existing systems thinking analysis methods. A summary of the findings is presented in Table 3.

## 5 Discussion

**Fig. 2:**
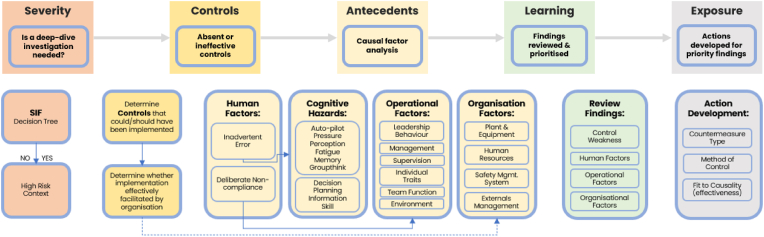
SCALE® process Model.

To advance change to systems thinking in the modern era, the authors are proposing a new way of assessing accident casualty for serious accident and fatality to address the current gap in methodology that exists using AcciMap, HFACS, STAMP-CAST and FRAM analysis models. Considering lessons learnt from the four foundational and contempory accident causation and incident models this paper is proposing a new and improved process model. The SCALE (Severity, Controls, Antecedents, Learning and Exposure) Process Model (Fig. 2) has been developed following a comprehensive review of the existing models, accident causation literature and continuous application pilot testing and refinement across a variety of organisational sectors and incidents. The fit for purpose SCALE Process Model addresses the need to upgrade incident reporting systems and analysis approaches [1]. The SCALE® Process Model is ideally situated to also address the need to apply a causation model across a variety of sectors and is not restricted to a single industry or incident.

The focus remains on the use of systemic methodologies, as systemic techniques provide a deeper understanding of how multiple factors within a system can contribute to an accident [20]. Underwood and Waterson (2019) also argue that despite the plethora of research about systemic techniques, this approach is still yet to gain widespread acceptance within the incident analytic community. The SCALE Process Model aims to assess organisational resources in order to respond to an incident, mitigate its impact and develop an understanding of the cause of the event in order to prevent it from occurring in the future.

The SCALE® Process Model is divided into five distinct sections across the continuum and can be applied to a single incident or a series of related incidents to affect organisational systemic change. The use of the SCALE acronym was intended to ensure that the five sections were considered and were easy to remember while acknowledging that there was a process to incident analysis. Recording the details around the ‘Severity’ of the incident requires a deep dive into the Serious Incident and Fatality (SIF) decision tree noting the extent of risk. The ‘Controls’ note absence or ineffectiveness of the controls, particularly Critical Controls, that could or should have been implemented. The ‘Antecedents’ (the thing that existed before or logically precedes another), consider the preceding event, condition or cause. The Antecedents section is divided into four areas, Human factors, Cognitive hazards, Operational factors and Organisational factors. The process model includes prompts in each section to help identify the elements of each of the antecedents that has contributed to the accident. The ‘Learning’ section reviews the findings and prioritises them according to significance. The learning section will help organisations identify the systemic changes that need to be addressed. Finally, ‘Exposure’ represents the actions that are developed following the prioritisation of the findings.

The SCALE® Process Model applies and prioritises a critical control lens which the other frameworks do not necessarily use. Incident Analytics® has developed a computerised analysis SAAS software program that inputs, assesses, reports and prioritises accident analysis findings. The software program has been modified and refined since 2018, incorporating the core elements of the four foundational analysis models (AcciMap, FRAM, STAMP and HFACS) while addressing the need to focus not simply on human/organisational error but on systemic organisational change. The generated report provides organisations with a comprehensive analysis of serious incidents and fatalities which can be used to inform future risk mitigation. The SCALE® Process Model addresses the limitations of the current four accident analysis methods which continue to focus on errors, malfunctions and deficiencies and can be highly complex and difficult to interpret. SCALE also addresses the gap in the ability to apply causation models across sectors. The SCALE® Process Models advanced computer analysis capability make it fit for purpose for the future of accident analysis.

## 6 Practical application of SCALE® process model

The SCALE® Process Model has been used as a causation analysis methodology within high-risk industrial organisations. Since its inception, SCALE has been used to analyse more than 10,000 incidents occurring in over 150 operating sites across Mining, Utilities, Agriculture and Transport industries since its inception. The analysis methodology has been used as a means of developing insight and learning to inform the following:

• Independent assessment of the governance of and classification of hi-potential incidents and reliability of incident reporting for boards
• Identification of the highest and most prevalent risk areas that benefit from increased critical control management.
• Develop strategy and risk management plans in response to serious injury and fatalities.
• Understand precursors and system factors that contribute to serious injury and fatality potential events.
• Development or strengthening of critical control performance standards as a byproduct of bow tie analysis.
• Enhancement of the design, management and supervision of high-risk work

## 7 Limitations

The rapid review was conducted using a systematic approach applying search terms consistently across multiple databases, however, there remains a variety of interchangeable terminology used to describe the application of accident analysis models which may have resulted in papers being missed. Reference list cross analysis was conducted to limit the impact of the terminology factor. Limited peer reviewed papers have been published in this field since 2018, despite this the five papers included in this review add to the information available on the application of analysis models and further informed the development of the SCALE® Process Model.

## 8 Conclusion

The development of systems thinking accident analysis methods over the last two decades has resulted in four main approaches, traditionally applied to a single industry or organisational sector. The most popular of these models is the HFACS model followed by AcciMap with STAMP and FRAM relatively new models with only minor application to date. It should be noted that all four of the current models continue to be difficult to apply across sectors and remain complex and difficult to interpret. The SCALE® Process Model incorporates all the essential elements of the four existing models but enables a wide range of application and systematic techniques to provide a deeper understanding of how each factor contributes to an accident. The SCALE® Process Model proposes a new innovative analysis software for the future of accident causation analysis.

Jpeg version.

Original version.

• Irrelevant to our review aims:
  • Wrong setting (*n* = 2)

## CRediT authorship contribution statement

**Joanne E. Porter:** Conceptualization, Methodology, Investigation, Formal analysis, Writing – original draft, Supervision, Writing – review & editing, Funding acquisition. **Elissa Dabkowski:** Conceptualization, Investigation, Formal analysis, Writing – review & editing. **Warren Smith:** Conceptualization, Investigation, Formal analysis, Writing – original draft, Writing – review & editing. **Alex Fernando:** Conceptualization, Investigation, Formal analysis, Writing – original draft, Writing – review & editing. **Liz Seaward:** Investigation, Formal analysis.

## Funding source

This literature review was funded by Incident Analytics Inc and conducted by the Collaborative Evaluation and Research Centre at 10.13039/501100006643Federation University Australia.

## A conflict of interest statement

The authors declare that they have no conflict of interest.
